# Supplementary figures and images for: The choice of the objective function in flux balance analysis is crucial for predicting replicative lifespans in yeast
Source: PLoS One. 2022 Oct 13;17(10):e0276112. doi: 10.1371/journal.pone.0276112 (PMC9560524; doi:10.1371/journal.pone.0276112)

## first objective

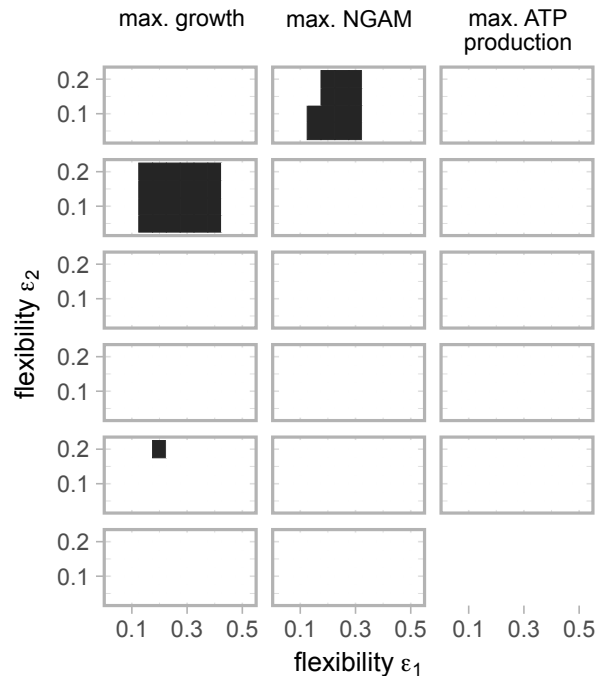

use  
parsimo-  
nious  
solution

## first objective

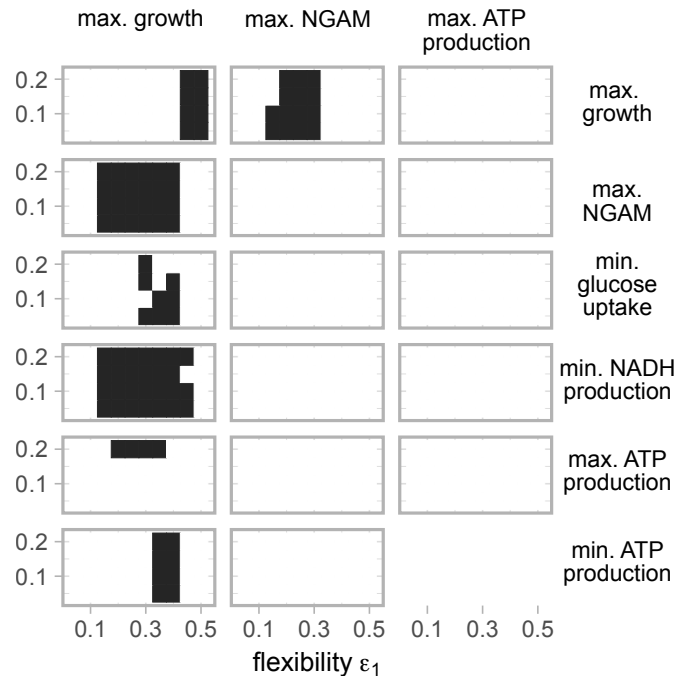

Supplement: S1 Fig — All parameters ϵ1,2 marked in black generate wildtype cells with a replicative lifespans between 20 and 30 divisions, and generation times between 1.5 and 2.3h, in our model. Based on Fig 1. (PDF) [file pone.0276112.s001.pdf]

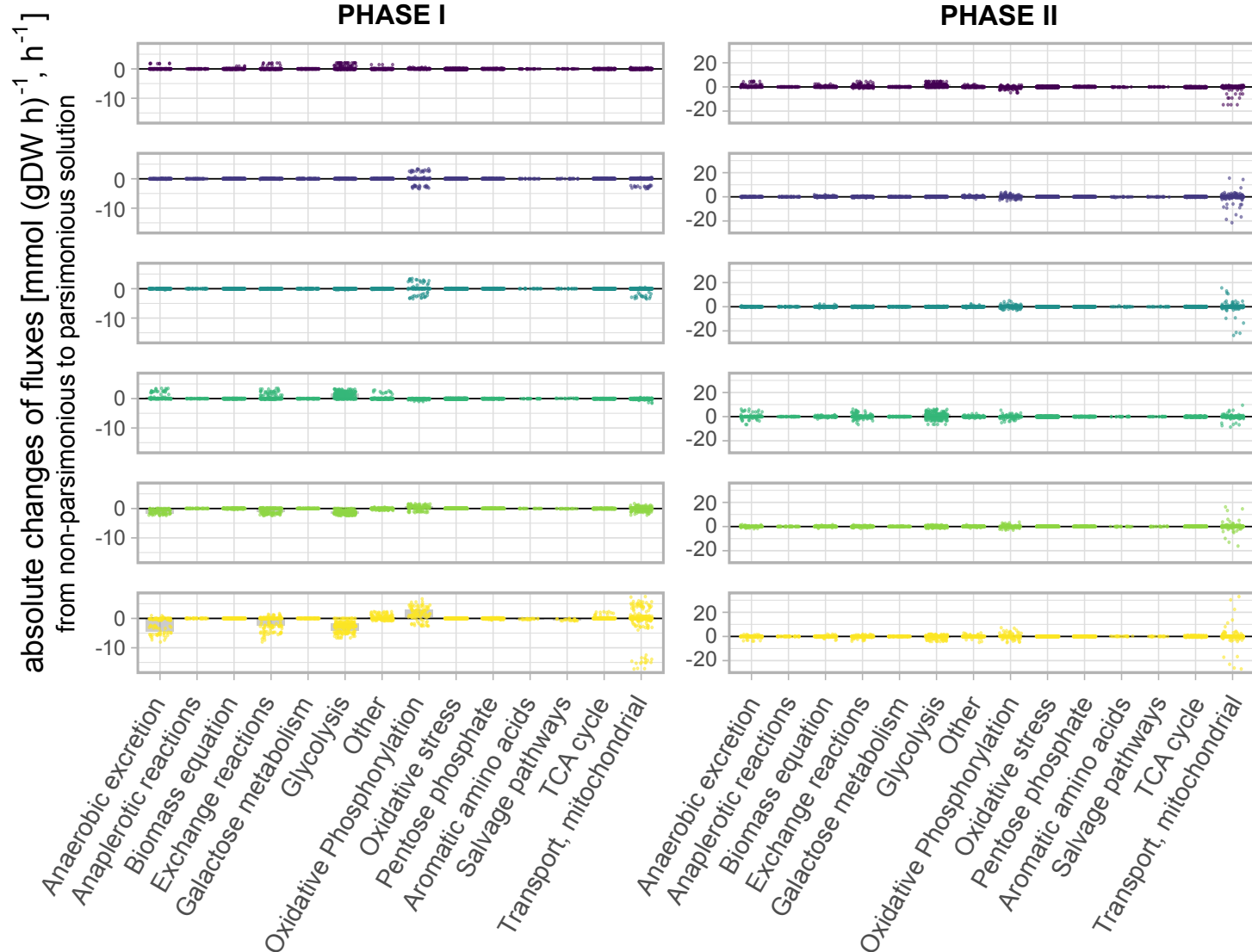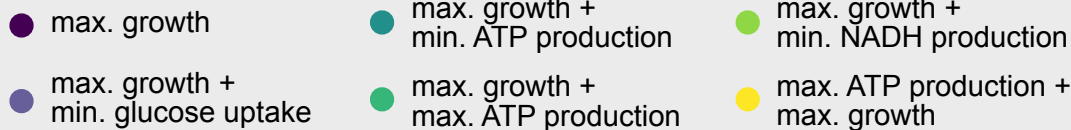

Supplement: S2 Fig — We limited the analysis to objectives that show a large increase in the replicative lifespans as a consequence of imposing parsimony. Included are 20 parameter combination with ϵ1 ≥ 0.3 and ϵ2 ≤ 0.2 per investigated objective (Fig 1). We averaged the fluxes over the two metabolic phases (left: I, right: II). The results are similar to Fig 2, but here each average flux is neither scaled by the glucose uptake rate, nor by the respective non-parsimonious flux, but is an absolute difference. (PDF) [file pone.0276112.s002.pdf]

## PHASE I

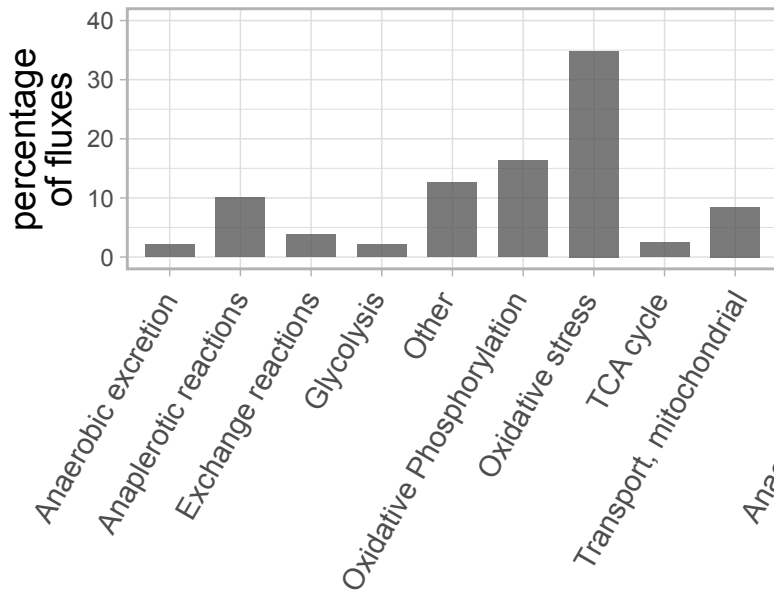

## PHASE II

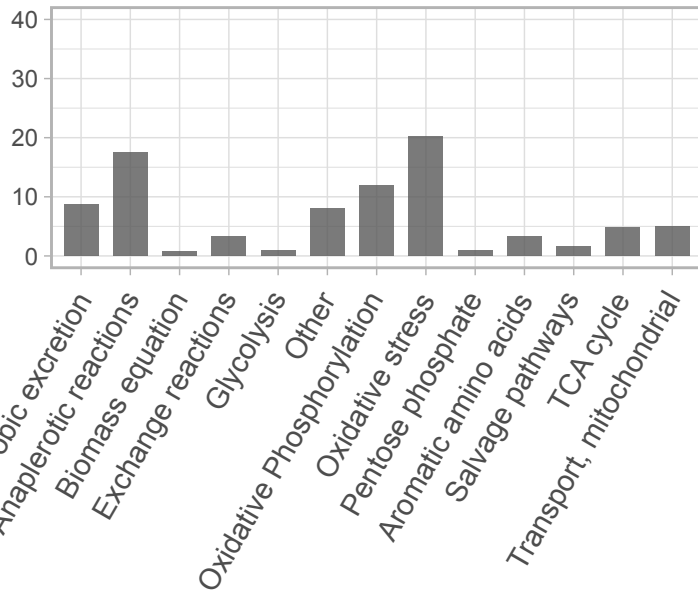

Supplement: S3 Fig — Percentage of fluxes in the respective pathways with relative change of at least 100% when imposing parsimony, being a subset of the fluxes shown in Fig 2B. All objective functions are merged in this plot. (PDF) [file pone.0276112.s003.pdf]
