# Supplementary material for: The choice of the objective function in flux balance analysis is crucial for predicting replicative lifespans in yeast
Source: PLoS One. 2022 Oct 13;17(10):e0276112. doi: 10.1371/journal.pone.0276112 (PMC9560524; doi:10.1371/journal.pone.0276112)
Supplement: S4 Fig — Relative changes of fluxes between the non-parsimonious and the parsimonious solution. Included are 20 parameter combination with ϵ1 ≥ 0.3 and ϵ2 ≤ 0.2 per investigated objective (Fig 1). We limited the analysis to objectives that show a large increase in the replicative lifespans as a consequence of imposing parsimony. Each flux is normalised by the glucose uptake rate and averaged over the metabolic phase (left: I, right: II). (PDF) [file pone.0276112.s004.pdf]

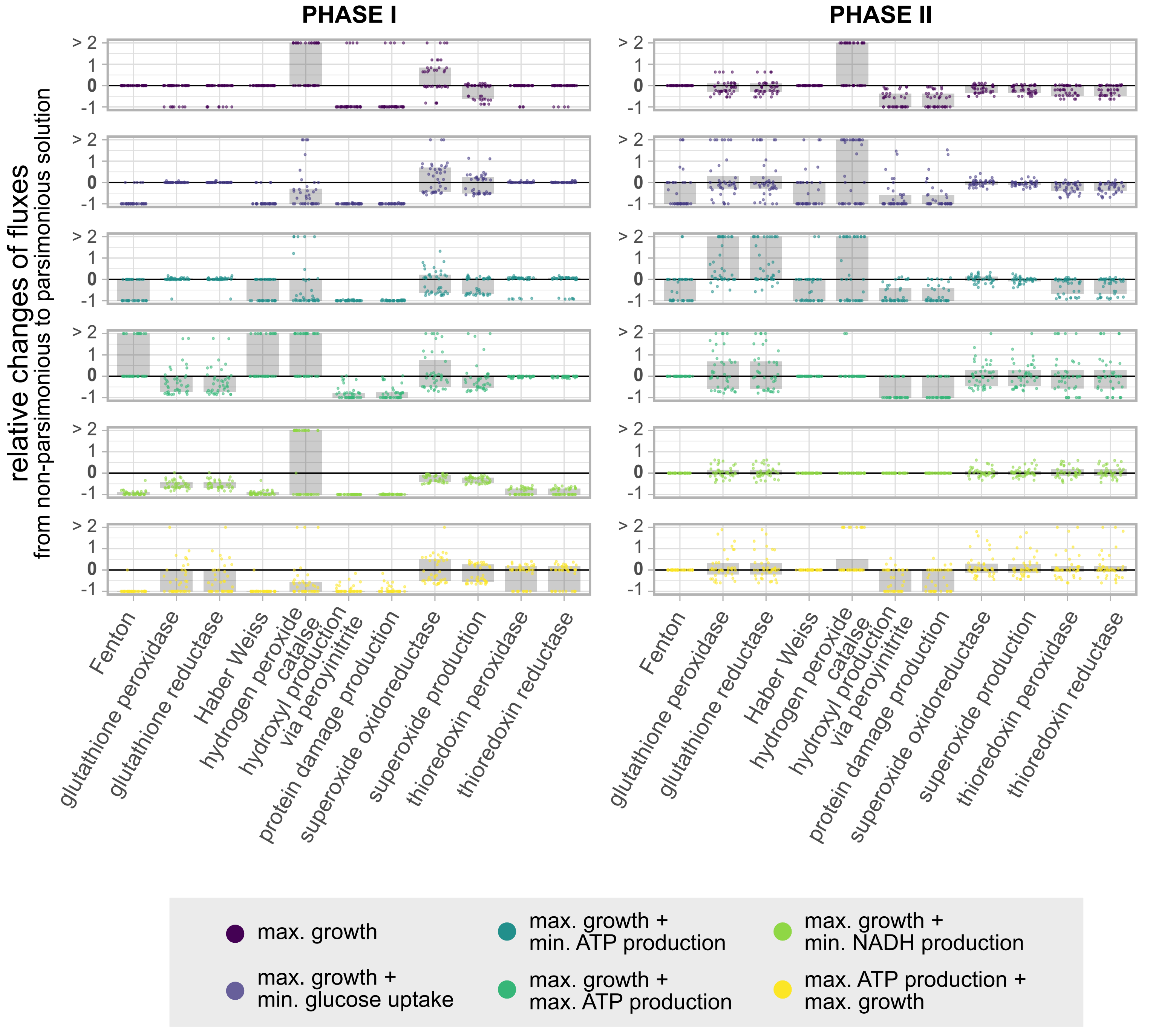

PHASE I
PHASE II
relative changes of fluxes
from non-parsimonious to parsimonious solution
> 2
1
0
-1
Fenton
glutathione peroxidase
glutathione reductase
Haber Weiss
hydrogen peroxide catalse
hydroxyl production via peroyinitrite
protein damage production
superoxide oxidoreductase
superoxide production
thioredoxin peroxidase
thioredoxin reductase
max. growth
max. growth + min. glucose uptake
max. growth + min. ATP production
max. growth + max. ATP production
max. growth + min. NADH production
max. ATP production + max. growth
