# Supplementary material for: The choice of the objective function in flux balance analysis is crucial for predicting replicative lifespans in yeast
Source: PLoS One. 2022 Oct 13;17(10):e0276112. doi: 10.1371/journal.pone.0276112 (PMC9560524; doi:10.1371/journal.pone.0276112)
Supplement: Attachment — Submitted filename: Plos objective function rebuttal.pdf [file pone.0276112.s005.pdf]

Comments to the Authors: Please note here if the review is uploaded as an attachment.

Reviewer #1: In this work, Schnitzer et al aimed to investigate the effect of the objective function in flux balance analysis (FBA) on yeast replicative aging. They analyzed how commonly used objectives in FBA affected evolutionarily important features in yeast cells.

The authors showed that the type of the objective function has a big say on yeast replicative lifespan as well as generation times, concluding that maximal growth was essential for replicative aging. Also, their findings indicated that using the parsimonious solution could lead to increased lifespan through rearrangement of fluxes.

Their model could reproduce close-to-experimental values for average replicative lifespan and generation times of wild-type yeast cells. The model also predicts features of replicative aging with distinct metabolic phases.

This is an interesting piece of work as it brings a novel way of thinking on the choice of the objective function by putting the objective in FBA in the context of evolutionary variables. The finding that maximal growth is the most important objective in regard to replicative lifespan will have significant impact.

We would like to thank the reviewer for a very careful and thorough evaluation of the manuscript. We have received very much appreciated, constructive and valuable comments and suggestions. We carefully addressed all comments which lead to a revision of the manuscript and the supplementary information.

I would ask the authors to revise their manuscript along the following two points:

1. Regarding their underlying multi-scale model, it would be good to explain/inform the reader about which parameters have been empirically known and which have not. Also, any quantitative assessment of the robustness of the overall model should ideally be made.

We provided a thorough description and motivation of all parameters in the linked paper that introduces the model (https://doi.org/10.1101/2022.03.07.483339). While the focus of this work is not the model, but how we used it to study the effect of the objective function on evolutionary observables such as lifespan, we here refer the reader to that paper instead, where all computational and mathematical details can be found, but also emphasised again the specific parameters that correspond to the reference cell (lines 280-285, and section Simulation Details).

2. There are previously-published yeast-aging-focused or time-dynamic single-cell-modeling-focused papers that should have been cited due to their relevance to the different sections of this manuscript:

http://doi.org/10.1016/j.celrep.2019.07.082
http://doi.org/10.1186/s12859-019-2921-3
http://doi.org/10.1186/s12918-015-0240-5

We disagree with the reviewer and do not find the publications relevant for this particular work concerning the objective function of the FBA model, while above mentioned paper are centered

around dynamic models. However, we acknowledge the relevance of those papers for the model itself and therefore included them in the linked publication (https://doi.org/10.1101/2022.03.07.483339).

Reviewer #2: The work by Schnitzer et al, evaluates the well-studied objective functions in Flux Balance Analysis, within the scope of replicative lifespan in yeast. The yeast model incorporates a boolean connection to gene network, thereby connecting transcriptome to metabolism. Owing additionally to the enzyme constraints, the model is taken as multi-omic. They find that the well-known and evolution-motivated maximum growth function works best for this case, with additional features when parsimonious solutions are considered. The article is written in a clear English with occasional overwordy explanations.

The model that the authors are using is the Yeast GSMM and they contextualize with a dynamic model to represent the replicative dynamics. The flux model is from an earlier work and the dynamic ODE model used to contextualize (replicative aging) is also from an earlier study of the same group focusing on trade-off between ATP demand and protein as driver for metabolic changes. The current paper reports the results of a systematic simulation results, while exploiting concepts related to multiobjective optimization, as sequential dual optimization, pareto front etc. The authors provide extensive discussion on the two parameters epsilon1 and 2 on describing, trade-off, when the objective function is switched, or the phase changes. There is no major concern on the methology used in the paper, neither for the models, nor for data/code sharing.

We would like to thank the reviewer for a very careful and thorough evaluation of the manuscript. We have received very much appreciated, constructive and valuable comments and suggestions. We carefully addressed all comments which lead to a revision of the manuscript and the supplementary information.

My main concern with this paper that it uses tools/models/concepts from earlier literature and the reported results are neither exciting nor surprising (importance of max growth has been well-discussed, motivated. The flux transition schemes from one state to another is similarly known). As such it seems like the paper is a simulation/reporting exercise. Despite that I find this work valuable, I think it stays below the high quality standards of PloS Computational Biology in terms of novelty and high impact.

In this work we explore the model we presented earlier (bioRxive https://doi.org/10.1101/2022.03.07.483339 and that received minor revision in the PloS Comp Biology (revision submitted), thus the novelty of the work presented here is not on the model itself (and we do not claim that in the ms).

Since, the connection between replicative life span (RLS) and growth rate is not intuitive, we now systemically show this. We look at the possible strategies the cell can use with the same objective function in order to reach wt or higher RLS.

Setting objective function in the context of ageing has not been done before and this could help in design optimal long-lived yeast relevant for industrial application. Previous studies only addressed this at the level of metabolism and young cells, but here, with the multi-scale model of yest replicative ageing, we developed previously, we have an opportunity to address these beyond metabolism and include mixed population. Thus, we look at the role of objective function in the multi-scale model of yeast replicative ageing, that includes an enzyme-constrained FBA model, a

Boolean model of nutrient signalling pathways and dynamic model of protein damage accumulation and cell growth.
