# Supplementary material for: The choice of the objective function in flux balance analysis is crucial for predicting replicative lifespans in yeast
Source: PLoS One. 2022 Oct 13;17(10):e0276112. doi: 10.1371/journal.pone.0276112 (PMC9560524; doi:10.1371/journal.pone.0276112)
Supplement: Attachment — Submitted filename: PLOS ONE REbuttal.pdf [file pone.0276112.s007.pdf]

Additional Editor Comments:
One major finding in the current version is that assuming maximal growth is essential for reaching realistic lifespans. Replicative lifespan have substantial natural variations, up to 200%. Growth and replicative lifespan are also influenced by growth media. So, we hope that this main conclusion is re-examined in the context of different genotypes and different growth conditions.
[Note: HTML markup is below. Please do not edit.]

We would like to thank the editor for taking the time to review our paper and for providing a careful and thorough evaluation of the manuscript.

This work is based on our multi-scale model of yest replicative ageing that has now been accepted for publication in PLoS Computational Biology (https://doi.org/10.1371/journal.pcbi.1010261). In that paper we observe the variation of replicative life span between 17 – 32 divisions. We are aware of natural variation of RLS, like those reported in Kaya et al 2021 (https://doi.org/10.7554/eLife.64860) or Janssens and Veenhoff 2016 (https://doi.org/10.1371/journal.pone.0167394). However, when it comes to ageing studies in yeast, standard laboratory strains show the RLS of typically 20-25 divisions, and this is the standard metrics used and we used this as a guidance for our studies. Further, in the work presented in this manuscript we use the same growth media that is a standard for FBA modeling in general.

We agree with the comment that many features are influenced by growth media, however, we aimed here to focus on standard media and examine typical objectives used in FBA community to make our work as applicable as possible for genome-scale modelers. Exploring those in the wider context is interesting, but this was not the aim of our study.

Reviewers' comments:

Reviewer's Responses to Questions
Comments to the Author

1. Is the manuscript technically sound, and do the data support the conclusions?

The manuscript must describe a technically sound piece of scientific research with data that supports the conclusions. Experiments must have been conducted rigorously, with appropriate controls, replication, and sample sizes. The conclusions must be drawn appropriately based on the data presented.
Reviewer #1: Partly

---

2. Has the statistical analysis been performed appropriately and rigorously?
Reviewer #1: Yes

---

3. Have the authors made all data underlying the findings in their manuscript fully available?

The PLOS Data policy requires authors to make all data underlying the findings described in their manuscript fully available without restriction, with rare exception (please refer to the Data Availability Statement in the manuscript PDF file). The data should be provided as part of the manuscript or its supporting information, or deposited to a public repository. For example, in addition to summary statistics, the data points behind means, medians and variance measures should be available. If there are restrictions on publicly sharing data—e.g. participant privacy or use of data from a third party—those must be specified.
Reviewer #1: Yes

4. Is the manuscript presented in an intelligible fashion and written in standard English?

PLOS ONE does not copyedit accepted manuscripts, so the language in submitted articles must be clear, correct, and unambiguous. Any typographical or grammatical errors should be corrected at revision, so please note any specific errors here.
Reviewer #1: Yes

5. Review Comments to the Author

Please use the space provided to explain your answers to the questions above. You may also include additional comments for the author, including concerns about dual publication, research ethics, or publication ethics. (Please upload your review as an attachment if it exceeds 20,000 characters)
Reviewer #1: In this work, Schnitzer et al aimed to investigate the relation between function in flux balance analysis on replicative lifespan (RLS) model of yeast aging. They developed a multi-scale model by incorporating a boolean connection to gene networks based on transcriptome and metabolome to analyze how commonly used objectives in FBA affected evolutionarily important cellular features in yeast cells to identify factors with significant impact on lifespan. Although there is some overlap with the previously published manuscript (https://doi.org/10.1101/2022.03.07.483339) from the same research group, this is an interesting study and would be of interest to the field.
Overall manuscript is well written. However, I have one major concern that needed to be addressed:

The study is based on simulation and their model could well predict the experimental observation for average RLS and generation times of WT yeast cells. This is a very important observation and needed to be further verified for other strains. There are publicly available resources of transcriptome, proteome and metabolome data for different yeast knock-out strains or interventions (e.g. TOR1 or caloric restriction). The model should be further tested whether it also predicts features of replicative aging in these strains/conditions along with RLS values.

We would like to thank the reviewer for taking the time to review our paper and for providing a careful and thorough evaluation of the manuscript.

The work presented here is based on our multi-scale model of yeast replicative ageing, which has been recently accepted for publication in PLoS Computational Biology (https://doi.org/10.1371/journal.pcbi.1010261). In this paper we have verified several of the model prediction (like deletion and overexpression, including the Snf1, Tor1 and Tor2 and many others) with publicly available data. Further, we were able to simulate transition of different metabolic phases and make predictions of enzyme perturbations that affect RLS. We do agree that model needs further testing, but that is outside the scope both of the published model and the work presented in this manuscript.

Minor concerns:
There should be more details for prediction of lifespan and growth time and prediction of increased lifespan. Since these are the major findings of the paper, it should be comprehensively described in method and discussed throughout the discussion section.
As far as I know, there is no single model that can predict yeast RLS with this much precision. So that this study will be in the high interest of the community. However, without testing/verifying their model in other strains/conditions, the validity and the significance of the findings would not be impactful.

Thanks for noticing the prediction power of the model when it comes to RLS. We believe that this clearly shows that to study ageing, holistic models are needed to be able to understand the synergistic effect of multiple processes affecting ageing. Previous ageing models focused only on individual processes, and this can be a reason why they lack the predictive power of our published model. In the paper where we introduced the multiscale model, we provide full details (model description, simulation details, predictions and validations). Similar to the above comment, we are aware that more validations are needed and is something our group is working with.
The aim of this paper submitted to PloS One and which is reviewed here is solely form the theoretical point of view on the role of the objective function, as common factor when it some to FBA models, but we investigate this in light of replicative lifespan. Using the correct objective in FBA to engineer “long-living” yeast could improve industrial titres and manufacturing of cell-based diagnostic devices.
We provide here the abbreviated summary of the model and full details of the optimization approach relevant for this study, together with the extensive simulation instructions and details on github.

---

6. PLOS authors have the option to publish the peer review history of their article (what does this mean?). If published, this will include your full peer review and any attached files.
