# Supplementary material for: The choice of the objective function in flux balance analysis is crucial for predicting replicative lifespans in yeast
Source: PLoS One. 2022 Oct 13;17(10):e0276112. doi: 10.1371/journal.pone.0276112 (PMC9560524; doi:10.1371/journal.pone.0276112)
Supplement: Attachment — Submitted filename: Review.pdf [file pone.0276112.s008.pdf]

In this work, Schnitzer et al aimed to investigate the effect of the objective function in flux balance analysis (FBA) on yeast replicative aging. They analyzed how commonly used objectives in FBA affected evolutionarily important features in yeast cells.

The authors showed that the type of the objective function has a big say on yeast replicative lifespan as well as generation times, concluding that maximal growth was essential for replicative aging. Also, their findings indicated that using the parsimonious solution could lead to increased lifespan through rearrangement of fluxes.

Their model could reproduce close-to-experimental values for average replicative lifespan and generation times of wild-type yeast cells. The model also predicts features of replicative aging with distinct metabolic phases.

This is an interesting piece of work as it brings a novel way of thinking on the choice of the objective function by putting the objective in FBA in the context of evolutionary variables. The finding that maximal growth is the most important objective in regard to replicative lifespan will have significant impact.

I would ask the authors to revise their manuscript along the following two points:

1. Regarding their underlying multi-scale model, it would be good to explain/inform the reader about which parameters have been empirically known and which have not. Also, any quantitative assessment of the robustness of the overall model should ideally be made.

2. There are previously-published yeast-aging-focused or time-dynamic single-cell-modeling-focused papers that should have been cited due to their relevance to the different sections of this manuscript:

   http://doi.org/10.1016/j.celrep.2019.07.082
   http://doi.org/10.1186/s12859-019-2921-3
   http://doi.org/10.1186/s12918-015-0240-5
