# Supplementary material for: The choice of the objective function in flux balance analysis is crucial for predicting replicative lifespans in yeast
Source: PLoS One. 2022 Oct 13;17(10):e0276112. doi: 10.1371/journal.pone.0276112 (PMC9560524; doi:10.1371/journal.pone.0276112)
Supplement: Attachment — Submitted filename: Rebuttal.pdf [file pone.0276112.s009.pdf]

The first study on the natural variation of yeast replicative lifespan was published in 2006, by Hong Qin, Meng Lu, "Natural variation in replicative and chronological life spans of Saccharomyces cerevisiae"

Experimental Gerontology, Volume 41, Issue 4,Pages 448-456.

The cell generation time (cell cycle) also increase close-to-linear during the aging process, as reported by Li et al, 2020 "A programmable fate decision landscape underlies single-cell aging in yeast", Science, v369, pp325-329. The Li 2020 paper is based on haploid cell.

In addition, wild isolate are diploid cells, are bigger and divide fast with a generation time about 45 minutes in early stage of aging. The reported 90 min lifespan in the literature are mostly based on haploid strains.

We would like to thank Editor for point out to those references. We have now incorporated them into the ms.

Below are the answers to the Reviewers comments attached as pdf. These are the comments from our original submission to PLoS Comp Bio and were already addressed when we transferred the ms to PLOS ONE.

Reviewer #1: In this work, Schnitzer et al aimed to investigate the effect of the objective function in flux balance analysis (FBA) on yeast replicative aging. They analyzed how commonly used objectives in FBA affected evolutionarily important features in yeast cells.

The authors showed that the type of the objective function has a big say on yeast replicative lifespan as well as generation times, concluding that maximal growth was essential for replicative aging. Also, their findings indicated that using the parsimonious solution could lead to increased lifespan through rearrangement of fluxes.

Their model could reproduce close-to-experimental values for average replicative lifespan and generation times of wild-type yeast cells. The model also predicts features of replicative aging with distinct metabolic phases.

This is an interesting piece of work as it brings a novel way of thinking on the choice of the objective function by putting the objective in FBA in the context of evolutionary variables. The finding that maximal growth is the most important objective in regard to replicative lifespan will have significant impact.

We would like to thank the reviewer for a very careful and thorough evaluation of the manuscript. We have received very much appreciated, constructive and valuable comments and suggestions. We

carefully addressed all comments which lead to a revision of the manuscript and the supplementary information.

I would ask the authors to revise their manuscript along the following two points:

1. Regarding their underlying multi-scale model, it would be good to explain/inform the reader about which parameters have been empirically known and which have not. Also, any quantitative assessment of the robustness of the overall model should ideally be made.

We provided a thorough description and motivation of all parameters in the linked paper that introduces the model (https://doi.org/10.1101/2022.03.07.483339). While the focus of this work is not the model, but how we used it to study the effect of the objective function on evolutionary observables such as lifespan, we here refer the reader to that paper instead, where all computational and mathematical details can be found, but also emphasised again the specific parameters that correspond to the reference cell (lines 280-285, and section Simulation Details).

2. There are previously-published yeast-aging-focused or time-dynamic single-cell-modeling-focused papers that should have been cited due to their relevance to the different sections of this manuscript:

http://doi.org/10.1016/j.celrep.2019.07.082
http://doi.org/10.1186/s12859-019-2921-3
http://doi.org/10.1186/s12918-015-0240-5

We disagree with the reviewer and do not find the publications relevant for this particular work concerning the objective function of the FBA model, while above mentioned paper are centered around dynamic models. However, we acknowledge the relevance of those papers for the model itself and therefore included them in the linked publication (https://doi.org/10.1101/2022.03.07.483339).
