# Supplementary material for: The choice of the objective function in flux balance analysis is crucial for predicting replicative lifespans in yeast
Source: PLoS One. 2022 Oct 13;17(10):e0276112. doi: 10.1371/journal.pone.0276112 (PMC9560524; doi:10.1371/journal.pone.0276112)
Supplement: Attachment — Submitted filename: Rebuttal.pdf [file pone.0276112.s010.pdf]

After communication with a reviewer, I would suggest you to briefly mention the following reference in your revised manuscript.

Song, R., Acar, M. Stochastic modeling of aging cells reveals how damage accumulation, repair, and cell-division asymmetry affect clonal senescence and population fitness. BMC Bioinformatics 20, 391 (2019). https://doi.org/10.1186/s12859-019-2921-3

I agree with the reviewer that Song19 is a relevant reference to your manuscript.

We have now incorporated this reference into the ms.
