# Supplementary material for: The choice of the objective function in flux balance analysis is crucial for predicting replicative lifespans in yeast
Source: PLoS One. 2022 Oct 13;17(10):e0276112. doi: 10.1371/journal.pone.0276112 (PMC9560524; doi:10.1371/journal.pone.0276112)
Supplement: Attachment — Submitted filename: Schnitzer et al., review_PlosOne.docx [file pone.0276112.s006.docx]

In this work, Schnitzer et al aimed to investigate the relation between function in flux balance analysis on replicative lifespan (RLS) model of yeast aging. They developed a multi-scale model by incorporating a boolean connection to gene networks based on transcriptome and metabolome to analyze how commonly used objectives in FBA affected evolutionarily important cellular features in yeast cells to identify factors with significant impact on lifespan. Although there is some overlap with the previously published manuscript (https://doi.org/10.1101/2022.03.07.483339) from the same research group, this is an interesting study and would be of interest to the field.

Overall manuscript is well written. However, I have one **major concern** that needed to be addressed:

The study is based on simulation and their model could well predict the experimental observation for average RLS and generation times of WT yeast cells. This is a very important observation and needed to be further verified for other strains. There are publicly available resources of transcriptome, proteome and metabolome data for different yeast knock-out strains or interventions (e.g. TOR1 or caloric restriction ). The model should be further tested whether it also predicts features of replicative aging in these strains/conditions along with RLS values.

**Minor concerns:**

There should be more details for prediction of lifespan and growth time and prediction of increased lifespan. Since these are the major findings of the paper, it should be comprehensively described in method and discussed throughout the discussion section.

As far as I know, there is no single model that can predict yeast RLS with this much precision. So that this study will be in the high interest of the community. However,without testing/verifying their model in other strains/conditions, the validity and the significance of the findings would not be impactful.
